# Drug exposure and risk factors of maculopathy in tamoxifen users

**DOI:** 10.1038/s41598-024-67670-x

**Published:** 2024-07-22

**Authors:** Hyeon Yoon Kwon, Jiyeong Kim, Seong Joon Ahn

**Affiliations:** 1 Department of Ophthalmology, Myongji Hospital, Hanyang University College of Medicine, 55, Hwasu-ro 14beon-gil, Deogyang-gu, Goyang-si, Gyeonggi-do 10475 Republic of Korea; 2 Department of Pre-Medicine, College of Medicine, and Biostatistics Laboratory, Medical Research Collaborating Center, Hanyang University, Seoul, Republic of Korea; 3 Department of Ophthalmology, Hanyang University Hospital, Hanyang University College of Medicine, 222-1 Wangsipli-Ro, Seongdong-Gu, Seoul, 04763 Republic of Korea

**Keywords:** Eye diseases, Risk factors

## Abstract

Tamoxifen, a pivotal therapy for hormone receptor-positive breast cancer, is known for its efficacy in reducing breast cancer recurrence and mortality. However, concerns about potential ocular complications, particularly maculopathy, have emerged. This study aims to investigate the risk and associated factors of diverse macular conditions in tamoxifen users, considering drug exposure, demographics, and systemic diseases. A nationwide cohort of tamoxifen users, comprised of 14,267 tamoxifen users, was analyzed using the health insurance review and assessment database in South Korea. Demographic and clinical characteristics were examined, and the cumulative incidence of macular diseases was stratified by age and cumulative tamoxifen dosage. We conducted logistic regression analysis to identify potential risk factors among clinical variables such as age, sex, indications for tamoxifen use, and systemic diseases associated with various macular conditions. Additionally, Cox proportional hazard models were used to determine the baseline clinical characteristics predictive of these macular conditions, with subsequent calculation of hazard ratios. Cumulative incidences of overall macular diseases, other maculopathy excluding common macular diseases, and macular edema were 26.4, 11.4, and 6.5%, respectively. The incidence of various macular conditions increased with age and the cumulative tamoxifen dose. Age, cumulative dose group, and liver diseases demonstrated significant associations with overall macular diseases and maculopathy excluding common macular diseases in multivariate logistic regression analyses (all *P* < 0.05). Furthermore, age emerged as significant predictive factors of maculopathy in Cox proportional hazard models. Tamoxifen-induced maculopathy poses a concern for prescribing physicians and ophthalmologists, and this study provides valuable insights into its risk and risk factors. This study may contribute to evidence-based guidelines for tamoxifen maculopathy screening, emphasizing the importance of considering age, cumulative dose, and liver diseases for recommendation on screening timing and frequency.

## Introduction

Tamoxifen, a selective estrogen receptor modulator, has long been a cornerstone in the treatment of hormone receptor-positive breast cancer^1,2^. Its efficacy in reducing the risk of recurrence and mortality in breast cancer patients is well established^3^. Guidelines from reputable organizations, such as the National Comprehensive Cancer Network (NCCN) and the American Society of Clinical Oncology (ASCO) provide recommendations regarding tamoxifen use in breast cancer treatment^4,5^. These guidelines suggest tamoxifen therapy in premenopausal women with a recommended daily dose of 20 mg. The therapy may last from 5 to 10 years depending on the patient's menopausal status and individual risk factors^6,7^. Furthermore, tamoxifen is used in metastatic breast cancer treatment, at recommended dosages ranging from 20 to 40 mg daily^8^.

Recent advancements in breast cancer management have led to the development of tailored treatment approaches, as evidenced by the Pan-Asian adapted ESMO Clinical Practice Guidelines^8^. These guidelines advocate for the inclusion of ovarian function suppression or ovarian ablation in conjunction with endocrine-based therapies for pre- and peri-menopausal women in Asian countries. Despite the well-established efficacy of tamoxifen and evidence-based recommendations or guidelines, the incorporation of these recommendations into national breast cancer guidelines varies across regions^7,9^. In Korea, however, the guidelines have limited specific directives on tamoxifen use for breast cancer treatment and prevention^9^.

However, the therapeutic advantages of tamoxifen may come with potential ocular complications, especially those impacting the macula^1,2,10–13^.The ocular side effects of tamoxifen, specifically tamoxifen-related retinopathy (maculopathy), have raised concerns among prescribing physicians and ophthalmologists. Alterations in the retina are reported to manifest in approximately 12% of individuals who have been using a daily dose of 20 mg of tamoxifen for a period exceeding two years^14^, corresponding to a cumulative dose of approximately 15 g. These alterations include the presence of crystalline deposits, telangiectasia, hyperreflective deposits in the inner retinal layers, pseudocystic foveal cavitation, and cystoid macular edema^1,2^. Understanding the demographic and clinical characteristics of individuals at a higher risk of developing maculopathy is crucial for identifying preventive measures and optimizing patient care. Only a few studies have focused on the risk and associated factors of maculopathy, mandating a comprehensive investigation into the risk factors and incidence rates of ocular complications associated with this widely used drug^1,14^.

In the present study, we investigated the risk of diverse macular conditions potentially associated with tamoxifen use in a nationwide cohort of tamoxifen users. By examining clinical characteristics, such as age, sex, and systemic diseases that potentially affect drug metabolism or retinal diseases, we explored the risk factors for macular conditions by focusing on age, drug exposure, and other systemic diseases. This study aimed to delineate the association between these factors and the occurrence of several types of maculopathies, providing valuable insights into the dose–response relationship between macular diseases and drug exposure and aiding in the formulation of evidence-based guidelines for tamoxifen maculopathy screening.

## Methods

### Study population

This study used the Health Insurance Review and Assessment (HIRA) database, a comprehensive repository encompassing health claims data of approximately 50 million individuals in South Korea^15,16^. This database, previously employed in a similar cohort study, contains information on diagnoses, procedures, prescriptions, visit dates, and demographic characteristics, utilizing codes from the Korean Standard Classification of Diseases, 7th and 8th Revision, with modifications from the International Statistical Classification of Diseases and Related Health Problems, Tenth Revision (ICD-10).

**Figure 1:**
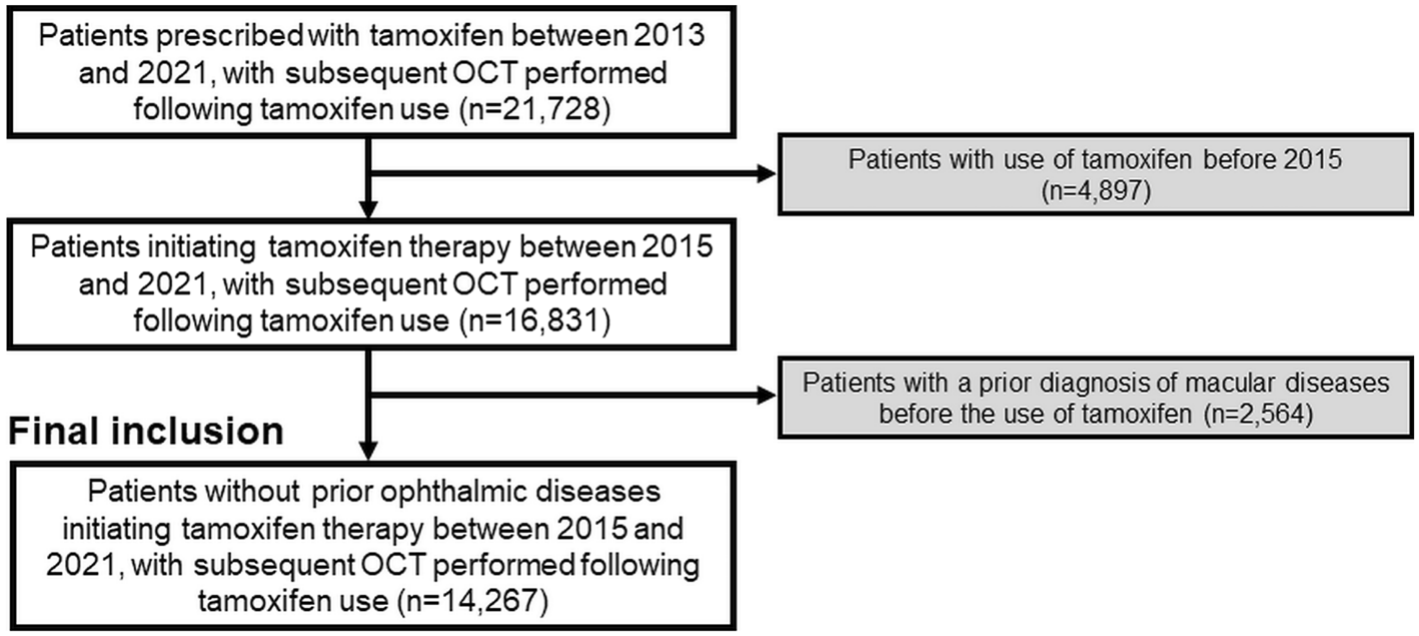
Flowchart illustrating the inclusion and exclusion criteria employed in this study, along with the populations after applying these criteria.

Tamoxifen users were identified within the database, focusing on individuals who initiated tamoxifen therapy between January 1, 2013, and December 31, 2021. To ensure accurate assessment of treatment duration, patients using tamoxifen before January 1, 2015, were excluded. Individuals with macular diseases before initiating tamoxifen treatment were also excluded aiming to include only patients with macular conditions occurring after tamoxifen initiation. The detailed inclusion/exclusion criteria and the number of subjects included/excluded are presented in Fig. 1. The Institutional Review Board of Hanyang University Hospital approved the study (IRB File No. 2023-01-003), which was conducted in accordance with the Declaration of Helsinki. The need for informed consent was waived by the Institutional Review Board of Hanyang University Hospital because of the retrospective nature of the study and the use of deidentified data. The reporting adhered to the Strengthening the Reporting of Observational Studies in Epidemiology (STROBE) guidelines.

### Definitions and evaluations

The identification and classification of macular conditions were based on specific KCD-8 codes within the dataset. Overall macular diseases were defined as the presence of H31.0 (macular scar), H35.3 (degeneration of the macula and posterior pole; including from H35.30 [nonexudative age-related macular degeneration] to H35.39 [unspecified macular degeneration]), or H35.80 (macular edema). Maculopathy excluding common macular diseases was delineated by the code H35.37 (toxic maculopathy) and H35.39 (unspecified macular degeneration; category for the macular diseases after exclusion of common macular diseases such as age-related macular degeneration and epiretinal membrane), while macular edema was specifically identified by the code H35.80 (macular edema).

The cumulative incidence of and risk factors for maculopathies among tamoxifen users were investigated. Among the patients receiving sufficient cumulative tamoxifen exposure, specifically 15 g in total (conventional daily dose of 20 mg for two years), the cumulative incidences of overall macular diseases, maculopathy excluding common macular diseases, and macular edema during the study period were evaluated. Although a consensus on the “at risk” population, targeted for toxicity screening, for tamoxifen maculopathy has not been reached, a common recommendation is to commence regular screenings including optical coherence tomography (OCT) for patients who have been on a tamoxifen dose of 20 mg/day for at least two years^2^, which is equivalent to a cumulative dose of 14.6 g. Therefore, we defined patients at risk as those with a cumulative tamoxifen dose ≥ 15 g. We calculated the incidences of several macular conditions according to age and cumulative dose groups among the patients at risk. These were stratified by cumulative dose and age categories into three cumulative dose groups (15 – < 30 g, 30 – < 45 g, and ≥ 45 g) and five age groups (below 40, 40–49, 50–59, 60–69, and ≥ 70).

Logistic regression analysis was performed to identify risk factors for diverse macular conditions among tamoxifen users. The analysis included demographic variables, such as sex and age, monitoring period, cumulative dose group, and systemic diseases affecting drug metabolism and the vascular system. We confirmed that variance inflation factors (VIF) were less than 10 and tolerance values were greater than 0.1 for all independent variables used for multivariate analyses, suggesting that multicollinearity was not a significant issue for multivariate analyses performed in this study. Thus, no further adjustment for statistical modeling was necessary^17–19^. Odds ratios (ORs) and their corresponding 95% confidence intervals (CI) are reported. Univariate and multivariate analyses were performed per macular condition to ascertain the individual impact of the identified risk factors while controlling for potential confounders. To identify predictive factors among the baseline clinical characteristics, Cox proportional hazard model regression was performed for demographic factors and systemic diseases, and hazard ratios (HR) were calculated for predictive factors.

Using the health claims data from the HIRA database, we assessed the tests performed for macular evaluation, including fundoscopy/fundus photography, OCT, and fluorescein angiography. The percentage of patients undergoing each test was assessed and presented as demographic and clinical characteristics.

### Data analysis

Descriptive statistics summarized the findings, presenting categorical variables as frequencies and percentages and continuous variables as mean (standard deviation) or median (interquartile range). Chi-square or Fisher’s exact tests were used to compare categorical variables between the groups. In our study, we used the Cochran–Mantel–Haenszel test, a statistical method for analyzing stratified or matched categorical data, to assess the association between a characteristic and a binary outcome, such as maculopathy status, while accounting for the effects of stratification and potential confounding factors.

The cumulative incidence of macular conditions was evaluated with Kaplan–Meier curves. Univariate and multivariate Cox proportional hazard models were utilized to identify predictive factors while controlling for potential confounding variables, including age, sex, and systemic diseases like diabetes mellitus, hypertension, hyperlipidemia, ischemic heart disease, and stroke. Statistical significance was set at *P* < 0.05. SAS Enterprise Guide version 7.1 conducted all analyses.

## Results

### Demographic and clinical characteristics of study population

Table 1 provides a comprehensive overview of the demographic and clinical characteristics of the tamoxifen users enrolled in this study, revealing the diverse profiles of individuals undergoing tamoxifen therapy. The cohort, consisting of 14,267 users, exhibited a notable sex distribution, with 79.9% female and 20.1% male. The mean age of the population was 54.0 years, with a range from 4 to 94 years. The most common age group among tamoxifen users included in this study was 40–49 years old, comprising 37.6% of the cohort. Underlying diseases including diabetes (30.8%), hypertension (37.7%), dyslipidemia (61.1%), kidney disease (16.1%), and liver disease (41.1%) were prevalent in the cohort. The primary indications for tamoxifen use varied, with breast cancer being the dominant indication, accounting for 58.7% of the cases. Additionally, the table presents the mean duration of tamoxifen use (33.1 ± 23.4 months) and the distribution across different duration categories. The cumulative daily dose of tamoxifen, an indicator of drug exposure, was delineated, with 40.5% of the users falling within the < 15 g category. The cumulative doses for the other patients ranged as follows; 15 to 30 g, 27.6% (n = 3931); 30 to 45 g, 29.9% (n = 4269); and > 45 g, 2.0% (n = 284).

**Table 1:** Demographic and clinical information of the tamoxifen users included in this study.

Characteristics	Overall users (n = 14,267)
Sex	
Male:female	2,874(20.1%):11,393(79.9%)
Mean age (± SD), years	54.0 ± 12.5 (range: 4–94)
< 20	27* (0.2%)
20–29	129 (0.9%)
30–39	961 (6.7%)
40–49	5,358 (37.6%)
50–59	3,272 (22.9%)
60–69	2,410 (16.9%)
≥ 70	2,110 (14.8%)
Systemic diseases	
Diabetes mellitus	4,394 (30.8%)
With related (systemic) complications	2,536 (17.8%)
Hypertension	5,374 (37.7%)
Dyslipidemia	8,711 (61.1%)
Hyperlipidemia	4,310 (30.2%)
Hypercholesterolemia	1,999 (14.0%)
Ischemic heart disease	1,426 (10.0%)
Stroke	599 (4.2%)
Kidney disease	2,302 (16.1%)
Liver disease	5,865 (41.1%)
Indication for tamoxifen use	
Breast cancer	8,373 (58.7%)
Ductal carcinoma in situ	1,849 (13.0%)
Gynecomastia	2,484 (17.4%)
Others	1,561 (10.9%)
Mean daily dose of tamoxifen (± SD), mg/day	19.9 ± 4.1
Less than 15 mg	672 (4.7%)
15–20 mg	379 (2.7%)
20–25 mg	12,909 (90.5%)
25 mg or greater	307 (2.2%)
Mean duration of tamoxifen use (± SD), months	33.1 ± 23.4
Less than 1 year	3,972 (27.8%)
1–2 years	1,645 (11.5%)
2–3 years	1,981 (13.9%)
3–4 years	1,890 (13.3%)
4–5 years	2,800 (19.6%)
5 years or longer	1,979 (13.9%)
Mean cumulative daily dose of tamoxifen (± SD), mg/day	19.8 ± 14.2
Less than 15 g	5,783 (40.5%)
15–30 g	3,931 (27.6%)
30–45 g	4,269 (29.9%)
45 g or greater	284 (2.0%)
Macular evaluation	
Fundus examination (funduscopy or fundus photography)	13,931 (97.6%)
Optical coherence tomography	14,267 (100%)
Fluorescein angiography	751 (5.3%)

SD, standard deviation.

*Includes 1 child (4-year-old) and 26 adolescent (11 to 19 years old) patients.

### Cumulative incidences of diverse macular conditions in overall patients and subgroups according to drug exposure level or age groups

**Figure 2:**
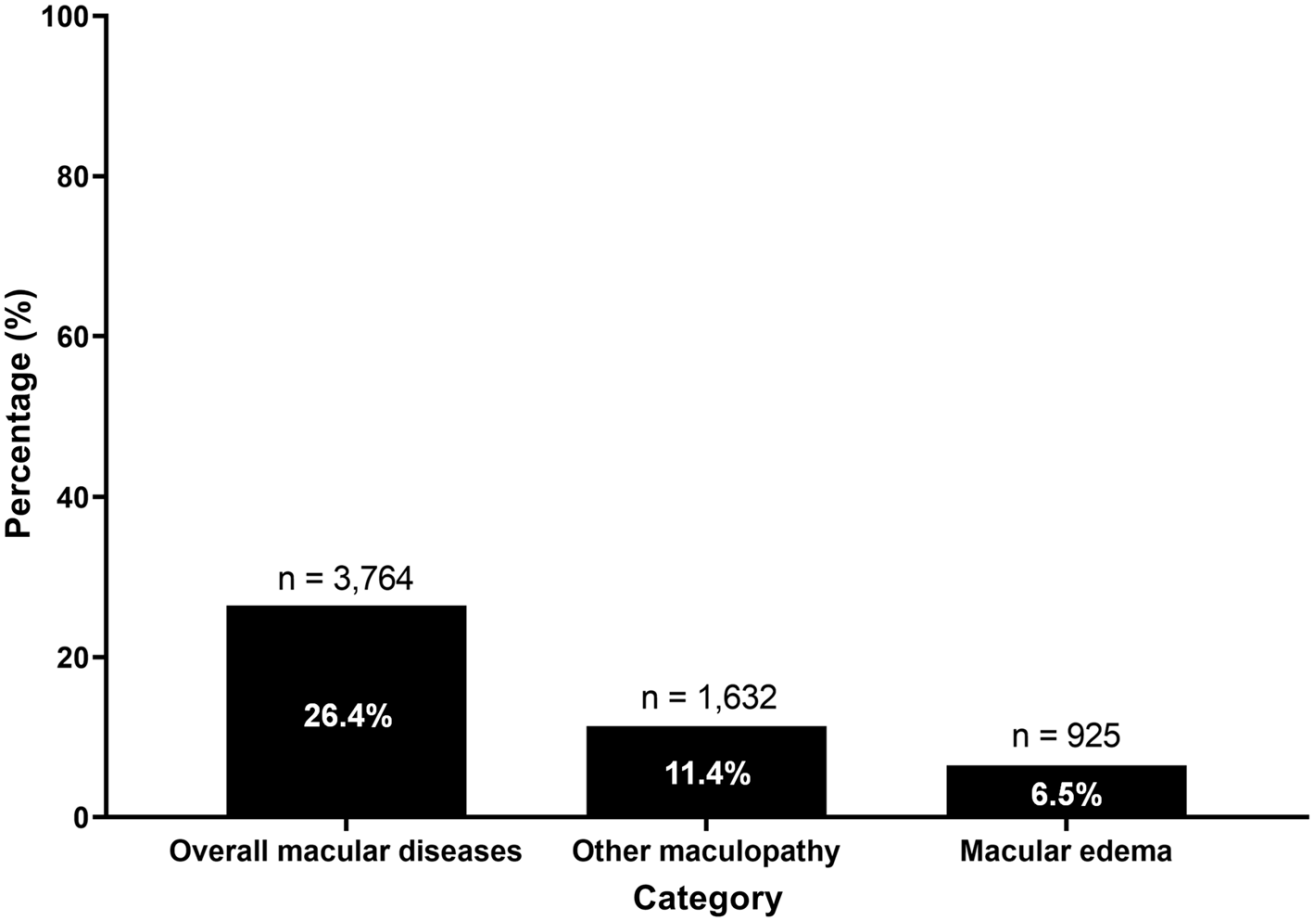
Cumulative incidences of macular conditions potentially affected by tamoxifen in the users.

Figure 2 shows the overall cumulative incidence of macular diseases (26.4%; n = 3,764), maculopathy excluding common macular diseases (11.4%; n = 1,632), and macular edema (6.5%; n = 925) in the entire patient cohort during the study period. However, common macular diseases such as age-related macular degeneration, epiretinal membrane, and macular hole were observed in 8.5% of the included patients over the study period.

Table 2 presents the cumulative incidence of overall macular diseases, other maculopathy excluding common macular diseases, and macular edema among the different risk groups of tamoxifen users during the study period, stratified by cumulative dose and age categories. For overall macular diseases, the cumulative incidence showed variations across both age and cumulative dose groups. Notably, a trend of increasing cumulative incidence was observed with higher age groups and cumulative tamoxifen doses (*P* < 0.001 by the Cochran–Mantel–Haenszel test), which was statistically significant. This pattern is consistent with maculopathy excluding common macular diseases and macular edema, highlighting the potential influence of age and cumulative tamoxifen dosage on the development of these macular conditions. The presented *P*-values (< 0.001 for both maculopathy excluding common macular diseases and macular edema) indicated statistically significant differences among the risk groups. These findings suggest the importance of considering both age and cumulative tamoxifen dosage when assessing the risk of overall macular diseases, maculopathy excluding common macular diseases, and macular edema in tamoxifen users.

**Table 2:** Cumulative incidences of overall macular diseases, unspecified maculopathy excluding common macular diseases, and macular edema in different cumulative dose groups among patients at risk (with tamoxifen exposure ≥ 15 g).

Cumulative dose group	Age					*P* value*
	< 40 (n = 695)	40–49 (n = 4,018)	50–59 (n = 2,090)	60–69 (n = 1,052)	≥ 70 (n = 629)	
Overall macular diseases						
15-30g	41 (12.9%)	353 (20.1%)	233 (23.8%)	193 (36.6%)	136 (39.0%)	< 0.001
30-45g	55 (16.3%)	447 (21.4%)	293 (27.4%)	211 (42.0%)	132 (48.4%)	
45g or greater	4 (9.8%)	42 (24.3%)	11 (26.8%)	13 (59.1%)	4 (57.1%)	
Maculopathy excluding common macular diseases						
15-30g	14 (4.4%)	160 (9.1%)	96 (9.8%)	86 (16.3%)	56 (16.1%)	< 0.001
30-45g	19 (5.6%)	184 (8.8%)	132 (12.4%)	106 (21.1%)	64 (23.4%)	
45g or greater	1 (2.4%)	16 (9.3%)	8 (19.5%)	4 (18.2%)	1 (14.3%)	
Macular edema						
15-30g	18 (5.7%)	93 (5.3%)	60 (6.1%)	37 (7.0%)	29 (8.3%)	< 0.001
30-45g	20 (5.9%)	131 (6.3%)	54 (5.1%)	45 (9.0%)	29 (10.6%)	
45g or greater	–	6 (3.5%)	1 (2.4%)	4 (18.2%)	–	

*Cochran-Mantel–Haenszel test.

### Risk factors and baseline predictive factors for maculopathy in tamoxifen users

Table 3 depicts the outcomes of the logistic regression analyses exploring the risk factors associated with various macular conditions among tamoxifen users. In the univariate analysis, several demographic and clinical characteristics were significantly associated with macular diseases. In multivariate analysis, after adjusting for potential confounders, advancing age was significantly associated with overall macular diseases and maculopathy excluding common macular diseases (both *P* < 0.001). Moreover, specific systemic diseases such as liver diseases emerged as significant risk factors across different macular conditions. The cumulative dose group was significantly associated with all three macular conditions (all *P* < 0.001). The group with a cumulative dose exceeding 45 g exhibited the highest OR for overall macular diseases (2.31, 95% CI 1.73–3.10) and maculopathy excluding common macular diseases (1.78, 95% CI 1.18–2.69). Additionally, liver disease was associated with overall macular diseases and maculopathy excluding common macular diseases, with ORs of 1.24 (95% CI 1.14–1.35) and 1.18 (95% CI 1.05–1.32), respectively. Subgroup analyses of risk factors for macular conditions among patients with breast cancer are presented in Supplementary Table 1. These results were similar to those observed in the overall population of tamoxifen users.

**Table 3:** Risk factors for several macular conditions on logistic regression analyses in tamoxifen users.

Factors	Overall macular diseases				Maculopathy excluding common macular diseases				Macular edema			
	Univariate		Multivariate		Univariate		Multivariate		Univariate		Multivariate	
	OR (95% CI)	*P*	OR (95% CI)	*P*	OR (95% CI)	*P*	OR (95% CI)	*P*	OR (95% CI)	*P*	OR (95% CI)	*P*
Sex*	0.63 (0.58–0.69)	< 0.001	0.93 (0.73–1.17)	0.524	0.72 (0.64–0.81)	< 0.001	1.17 (0.85–1.60)	0.343	0.69 (0.59–0.80)	< 0.001	0.86 (0.56–1.31)	0.471
Age	1.03 (1.03–1.04)	< 0.001	1.03 (1.03–1.03)	< 0.001	1.03 (1.03–1.04)	< 0.001	1.03 (1.03–1.04)	< 0.001	1.01 (1.01–1.02)	< 0.001	1.00 (1.00–1.00)	0.394
Monitoring period, months	0.99 (0.99–0.99)	< 0.001	0.99 (0.99–0.99)	< 0.001	1.00 (0.99–1.00)	< 0.001	0.99 (0.99–1.00)	< 0.001	0.99 (0.99–1.00)	< 0.001	0.99 (0.99–0.99)	< 0.001
Indications for tamoxifen use^†^		< 0.001		0.051		< 0.001		0.012		< 0.001		0.154
Ductal carcinoma in situ	1.16 (1.03–1.30)		1.02 (0.90–1.14)		1.20 (1.02–1.40)		1.04 (0.88–1.22)		0.90 (0.72–1.11)		0.85 (0.68–1.05)	
Gynecomastia	1.66 (1.51–1.83)		1.38 (1.08–1.76)		1.50 (1.31–1.71)		1.47 (1.06–2.05)		1.45 (1.23–1.71)		1.29 (0.84–2.00)	
Others	1.23 (1.09–1.38)		1.14 (1.00–1.31)		1.39 (1.18–1.63)		1.31 (1.10–1.56)		0.92 (0.73–1.16)		0.89 (0.70–1.14)	
Cumulative dose group^‡^		0.004		< 0.001		0.148		< 0.001		0.113		< 0.001
15-30g	0.84 (0.77–0.93)		1.31 (1.17–1.47)		0.87 (0.77–0.99)		1.23 (1.05–1.43)		0.87 (0.74–1.03)		1.20 (0.98–1.47)	
30-45g	0.95 (0.87–1.04)		1.89 (1.67–2.13)		1.00 (0.88–1.13)		1.63 (1.39–1.93)		0.95 (0.81–1.11)		1.59 (1.28–1.96)	
45g or greater	0.92 (0.71–1.21)		2.31 (1.73–3.10)		0.88 (0.60–1.29)		1.78 (1.18–2.69)		0.55 (0.30–1.01)		1.00 (0.53–1.89)	
Diabetes	1.33 (1.23–1.44)	< 0.001	0.94 (0.83–1.06)	0.298	1.23 (1.11–1.37)	< 0.001	1.02 (0.87–1.20)	0.769	1.39 (1.21–1.60)	< 0.001	0.96 (0.77–1.20)	0.705
Hypertension	1.58 (1.47–1.70)	< 0.001	1.10 (1.00–1.21)	0.055	1.52 (1.37–1.68)	< 0.001	1.08 (0.95–1.23)	0.231	1.35 (1.18–1.54)	< 0.001	1.13 (0.95–1.33)	0.172
Hyperlipidemia	1.38 (1.28–1.50)	< 0.001	1.10 (0.98–1.23)	0.125	1.37 (1.23–1.53)	< 0.001	1.14 (0.97–1.33)	0.110	1.28 (1.11–1.47)	0.001	1.05 (0.86–1.29)	0.624
Ischemic heart disease	1.50 (1.34–1.69)	< 0.001	0.98 (0.86–1.12)	0.739	1.22 (1.04–1.44)	0.015	0.83 (0.69–0.99)	0.037	1.28 (1.04–1.57)	0.020	0.93 (0.74–1.17)	0.524
Stroke	1.56 (1.31–1.85)	< 0.001	1.03 (0.86–1.12)	0.753	1.67 (1.34–2.08)	< 0.001	1.22 (0.97–1.54)	0.096	1.27 (0.94–1.72)	0.121	0.97 (0.71–1.34)	0.863
Kidney disease	1.34 (1.21–1.48)	< 0.001	1.06 (0.95–1.18)	0.315	1.20 (1.05–1.37)	0.009	0.99 (0.86–1.15)	0.911	1.25 (1.05–1.48)	0.010	1.02 (0.84–1.23)	0.859
Liver disease	1.32 (1.22–1.42)	< 0.001	1.24 (1.14–1.35)	< 0.001	1.27 (1.15–1.41)	< 0.001	1.18 (1.05–1.32)	0.006	1.22 (1.07–1.40)	0.003	1.15 (0.99–1.33)	0.072

*Male as reference.

^**†**^Breast cancer as reference.

^‡^Less than 15g as reference.

Table 4 presents the HR with 95% CI and the corresponding P-values obtained by the Cox proportional hazard model for several demographic and systemic factors at baseline that contribute to the development of several macular conditions in tamoxifen users. The multivariate analysis revealed a significant association between sex and maculopathy excluding common macular diseases (*P* = 0.009). Age was also identified as a substantial risk factor for all three macular conditions, emphasizing the heightened vulnerability of older individuals to the development of associated macular diseases (all *P* < 0.05). Systemic diseases, including diabetes and hyperlipidemia, were not associated with any of the three conditions. Additionally, kidney or liver disease did not show a statistically significant association with any of the macular conditions in the Cox multivariate regression analyses (all *P* > 0.05).

**Table 4:** Baseline characteristics predictive of various macular conditions and their hazard ratios (HR) among overall tamoxifen users.

Factors	Overall macular diseases				Maculopathy excluding common macular diseases				Macular edema			
	Univariate		Multivariate		Univariate		Multivariate		Univariate		Multivariate	
	HR (95% CI)	*P*	HR (95% CI)	*P*	HR (95% CI)	*P*	HR (95% CI)	*P*	HR (95% CI)	*P*	HR (95% CI)	*P*
Sex*	0.65 (0.60–0.70)	< 0.001	1.21 (1.00–1.46)	0.051	0.70 (0.62–0.78)	< 0.001	1.48 (1.11–1.98)	0.009	0.66 (0.57–0.77)	< 0.001	1.04 (0.70–1.56)	0.834
Age	1.03 (1.03–1.03)	< 0.001	1.03 (1.03–1.03)	< 0.001	1.03 (1.03–1.04)	< 0.001	1.03 (1.03–1.04)	< 0.001	1.01 (1.01–1.02)	< 0.001	1.01 (1.00–1.01)	0.012
Indications for tamoxifen use^†^		< 0.001		0.669		< 0.001		0.617		< 0.001		0.075
Ductal carcinoma in situ	1.15 (1.04–1.26)		1.04 (0.94–1.15)		1.19 (1.03–1.39)		1.07 (0.92–1.24)		0.91 (0.74–1.12)		0.88 (0.71–1.08)	
Gynecomastia	1.59 (1.47–1.73)		1.00 (0.82–1.22)		1.54 (1.36–1.74)		1.15 (0.85–1.56)		1.50 (1.28–1.75)		0.91 (0.60–1.39)	
Others	1.14 (1.03–1.27)		0.96 (0.86–1.07)		1.28 (1.10–1.49)		1.09 (0.92–1.28)		0.89 (0.71–1.11)		0.74 (0.58–0.95)	
Diabetes	1.17 (1.04–1.19)	0.001	0.95 (0.79–1.14)	0.574	1.03 (0.93–1.14)	0.568	0.90 (0.68–1.20)	0.470	1.20 (1.05–1.37)	0.009	0.90 (0.64–1.28)	0.558
Hypertension	1.36 (1.27–1.45)	< 0.001	1.01 (0.93–1.09)	0.884	1.34 (1.21–1.47)	< 0.001	0.99 (0.88–1.12)	0.905	1.23 (1.08–1.40)	0.002	1.03 (0.88–1.21)	0.717
Hyperlipidemia	1.12 (1.04–1.19)	0.002	0.97 (0.90–1.04)	0.426	1.11 (1.00–1.23)	0.042	0.98 (0.87–1.09)	0.659	1.07 (0.94–1.23)	0.311	0.96 (0.82–1.11)	0.540
Ischemic heart disease	1.25 (1.14–1.38)	< 0.001	0.90 (0.81–1.00)	0.051	1.06 (0.92–1.24)	0.420	0.78 (0.66–0.92)	0.004	1.14 (0.93–1.38)	0.202	0.86 (0.69–1.08)	0.194
Stroke	1.28 (1.11–1.46)	0.001	0.89 (0.77–1.03)	0.129	1.34 (1.10–1.63)	0.004	1.00 (0.81–1.23)	0.988	1.11 (0.83–1.49	0.467	0.85 (0.63–1.15)	0.284
Kidney disease	1.08 (1.00–1.17)	0.061			0.97 (0.86–1.10)	0.662			1.05 (0.89–1.23)	0.596		
Liver disease	0.98 (0.92–1.05)	0.583			0.95 (0.86–1.05)	0.297			0.94 (0.83–1.07)	0.359		

*Male as reference.

^**†**^Breast cancer as reference.

## Discussion

Tamoxifen, a vital component in the treatment of hormone receptor-positive breast cancer, has long been recognized for its efficacy in reducing cancer recurrence and mortality^3^. However, because the ocular side effects of tamoxifen, particularly tamoxifen maculopathy, present a critical concern in clinical practice for tamoxifen users^1,2^, our study investigated the risk of macular conditions potentially associated with tamoxifen toxicity. From a comprehensive analysis of the demographic and clinical characteristics of the tamoxifen user cohort, our findings on the factors influencing the risk of maculopathy may contribute to the growing body of knowledge concerning the risk and risk factors of maculopathy in tamoxifen users, a topic not extensively studied.

Tamoxifen retinopathy can present in several forms^10–14,20–23^, and similarities with macular telangiectasia, type 2, have been noted in previous studies^1,24^. Therefore, the macular abnormalities observed on OCT in tamoxifen users may be recognized as a different condition, rather than tamoxifen maculopathy. Furthermore, this maculopathy has not been adequately recognized by ophthalmologists due to limited awareness; thus, it is likely that it has been misdiagnosed as other macular conditions such as macular degeneration and macular edema than toxic maculopathy. To maximize capturing potential macular conditions associated with tamoxifen toxicity, we included several macular conditions for the outcomes, while excluding patients with such conditions prior to tamoxifen use. The prevalence of toxic maculopathy in our cohort was < 0.1%, which is significantly lower than previously reported rates^1,14^. This finding supports our approach to mitigate the risk of underestimating tamoxifen-related macular conditions.

The cumulative incidence in previous studies varied from 0.9% to 12%, largely depending on the cumulative dose in the study population^1,14^. Furthermore, macular diseases generally occur in elderly individuals, indicating the susceptibility of older patients to macular diseases. Recent studies have reported higher incidence rates than previous studies, which might be partly explained by advances in retinal imaging and its increased sensitivity in detecting microscopic retinal changes in tamoxifen users. Our study, including the patients undergoing OCT imaging, showed maculopathy excluding common macular diseases, the most specific category for tamoxifen maculopathy among our outcomes, in 11% of overall tamoxifen users. This was comparable to the recent figure of 12% among patients receiving tamoxifen^14^.

The cumulative incidences of overall macular diseases, maculopathy excluding common macular diseases, and macular edema, stratified by cumulative dose and age categories, provide valuable insights into the dose–response relationship between drug exposure and maculopathy incidence and age-related susceptibility. The statistical significance of these trends further strengthens the evidence of the impact of these factors on maculopathy risk. The increasing cumulative incidence observed in older age groups and with higher cumulative tamoxifen doses highlights the relevance of age and cumulative tamoxifen dosage in the development of macular conditions. This underscores the need to simultaneously consider both factors in maculopathy screening, suggesting that future guidelines should incorporate these aspects when formulating screening recommendations.

Our study investigated the risk factors of macular conditions among tamoxifen users, highlighting an important yet underexplored aspect of tamoxifen retinopathy. Our findings underscore the significance of age, cumulative tamoxifen dose, and liver diseases in maculopathy development, with older individuals and those with higher cumulative doses or liver diseases exhibiting heightened susceptibility. The observed associations between macular diseases and demographic and systemic factors emphasize the multifactorial nature of tamoxifen-induced maculopathy. These results have implications for clinical practice, suggesting the need for vigilant monitoring and tailored screening strategies, particularly for older patients and those with high exposure or liver diseases. Our study contributes to the growing body of evidence regarding tamoxifen-induced ocular complications, laying the groundwork for the development of evidence-based guidelines for maculopathy screening in high-risk tamoxifen users.

Liver diseases were significantly associated with both overall macular diseases and maculopathy, excluding common macular diseases. Furthermore, we noted a severity-dependent relationship between the severity groups (no liver disease, liver disease without liver cirrhosis or failure, and liver cirrhosis or failure) and the risk of overall macular diseases and maculopathy excluding common macular diseases (Supplementary Table 2). These findings suggest that the interaction effects of liver diseases on tamoxifen maculopathy may be due to the fact that tamoxifen is primarily excreted by the liver and biliary tract, indicating that liver disease may influence systemic tamoxifen concentration. However, liver function could not be assessed in this study, which limited our ability to draw conclusions regarding the true association between liver function and tamoxifen maculopathy. Hyperlipidemia was not significantly associated with macular conditions, although a recent study identified hypercholesterolemia as a risk factor for tamoxifen maculopathy^14^.

Our findings have important clinical implications for the management of patients with breast cancer receiving tamoxifen therapy in South Korea. This comprehensive analysis of the risk factors and cumulative incidences of diverse macular conditions associated with tamoxifen use provides valuable insights that can guide clinical practice and patient care. First, our key findings highlight the need for vigilant monitoring and screening of patients with breast cancer receiving tamoxifen, particularly those at higher risk for maculopathy, such as those with liver diseases (Supplementary Table 1). The observed dose–response relationship between cumulative tamoxifen exposure and the incidence of macular diseases and increased susceptibility of older patients underscores the importance of tailoring screening recommendations based on these risk factors. Incorporating these aspects into national guidelines for tamoxifen-induced maculopathy screening would enable clinicians to identify high-risk patients and implement timely interventions to mitigate the development of vision-threatening complications by early detection and management.

Moreover, this study revealed baseline predictive factors such as older age and female sex. For patients initiating tamoxifen therapy, it is crucial to carefully assess and consider these factors when planning maculopathy screening. Those with advanced age should undergo timely initial monitoring by ophthalmologists through better referral from prescribing physicians. By offering insights into the risk of several macular conditions and predictive factors in the Korean population, this study might provide a foundation for developing evidence-based guidelines on the frequency and timing of screening aimed at enhancing the ocular safety of tamoxifen use in breast cancer treatment in Korea. These findings can inform the creation of targeted screening programs and patient education efforts, ultimately improving the quality of care and visual outcomes for breast cancer patients undergoing this essential therapy.

While our study provides valuable insights into the risk factors and cumulative incidence of tamoxifen-induced maculopathy, certain limitations must be acknowledged to ensure a nuanced interpretation of the findings. The retrospective nature of our study, which relied on the HIRA database, introduced some inherent limitations. The identification and classification of macular conditions were based on specific KCD-7 or 8 codes within the dataset, potentially leading to misclassification or under-reporting. The operational definitions used for the inclusion and exclusion criteria may not have captured all relevant cases, thus contributing to selection bias. The absence of direct clinical assessments and the reliance on diagnostic codes may result in an incomplete representation of tamoxifen maculopathy status.

Moreover, we acknowledge the possibility of unmeasured confounding factors that were not accounted for in our analysis. Lifestyle factors, genetic predisposition, and other medications that could influence the development of maculopathy were not comprehensively considered in our study. Furthermore, the generalizability of our findings may be limited because this study focused exclusively on the South Korean population. Healthcare systems and patient demographics vary across regions, and caution should be exercised when extending our results to other populations with different characteristics. We could not obtain details on breast cancer from the database, which may affect the risk of tamoxifen toxicity in users. Additionally, mortality is an important competing risk factor for tamoxifen retinopathy, as it precludes the occurrence of the primary event of interest, retinal toxicity. However, 5-year mortality of breast cancer is 93.6% in Korea^25^, mortality may not be a significant competing risk, as the vast majority of tamoxifen users with breast cancer survive conventional tamoxifen therapy. However, future studies with detailed information on breast cancer and mortality are required for tamoxifen users to exclude mortality from the risks of tamoxifen retinopathy.

Despite these limitations, our study provides a robust exploration of the risk factors and cumulative incidence of diverse macular conditions among tamoxifen users at the national level. The insights gained from this study have implications for clinical practice by guiding the formulation of evidence-based guidelines for tamoxifen-induced maculopathy screening. The intricate interplay between age, cumulative tamoxifen dosage, and systemic diseases highlighted in this study serves as a foundation for future research and enhances our understanding of the ocular complications associated with this widely prescribed breast cancer therapy. Future research could expand its scope to investigate tamoxifen maculopathy in diverse ethnic groups by incorporating more comprehensive clinical assessments aimed at identifying tamoxifen-induced maculopathy, which would enhance the generalizability and validity of our findings.

### Supplementary Information

Supplementary Information.

## Data Availability

The datasets generated during and/or analyzed during the current study are available from the corresponding author on reasonable request.
