# Supplementary material for: Drug exposure and risk factors of maculopathy in tamoxifen users
Source: Sci Rep. 2024 Jul 22;14:16792. doi: 10.1038/s41598-024-67670-x (PMC11263388; doi:10.1038/s41598-024-67670-x)
Supplement: Supplementary file 1 — Supplementary Information. [file 41598_2024_67670_MOESM1_ESM.docx]

**Supplementary Online Content**

**Supplemental Table 1.** Risk factors for several macular conditions on logistic regression analyses in tamoxifen users with breast cancer (n=8,373)

**Supplemental Table 2.** Association between liver disease severity and the risk (odds ratio [OR]) of overall macular diseases and maculopathy excluding common macular diseases, adjusted for sex, age, and cumulative dose group.

This supplementary material has been provided by the authors to give readers additional information about their work.

**Supplementary Table 1.** Risk factors for several macular conditions on logistic regression analyses in tamoxifen users with breast cancer (n=8,373)

| **Factors** | **Overall macular diseases** | | | | **Maculopathy excluding common macular diseases** | | | | | | **Macular edema** | | | |
| --- | --- | --- | --- | --- | --- | --- | --- | --- | --- | --- | --- | --- | --- | --- |
|  | **Univariate** | | **Multivariate** | | **Univariate** | | **Multivariate** | | | | **Univariate** | | **Multivariate** | |
|  | **OR (95% CI)** | **P** | **OR (95% CI)** | **P** | **OR (95% CI)** | **P** | | **OR (95% CI)** | **P** | | **OR (95% CI)** | **P** | **OR (95% CI)** | **P** |
| **Sex*** | 0.65  (0.44-0.96) | 0.029 | 1.14  (0.76-1.71) | 0.518 | 0.73  (0.43-1.25) | 0.250 | |  | |  | 0.65  (0.35-1.22) | 0.183 |  |  |
| **Age** | 1.04  (1.03-1.04) | <0.001 | 1.04  (1.03-1.05) | <0.001 | 1.03  (1.03-1.04) | <0.001 | | 1.04  (1.03-1.04) | | <0.001 | 1.01  (1.00-1.02) | 0.003 | 1.01  (1.00-1.01) | 0.293 |
| **Monitoring period, months** | 0.99  (0.99-0.99) | <0.001 | 0.99  (0.99-0.99) | <0.001 | 1.00  (0.99-1.00) | <0.001 | | 0.99  (0.99-1.00) | | <0.001 | 0.99  (0.99-1.00) | <0.001 | 0.99  (0.99-1.00) | <0.001 |
| **Cumulative dose group^†^** |  | 0.019 |  | <0.001 |  | 0.288 | |  | | <0.001 |  | 0.323 |  |  |
| 15-30g | 1.06  (0.93-1.22) |  | 1.34  (1.16-1.54) |  | 1.07  (0.89-1.30) |  | | 1.28  (1.05-1.56) | |  | 1.06  (0.84-1.34) |  |  |  |
| 30-45g | 1.21  (1.06-1.38) |  | 2.02  (1.73-2.35) |  | 1.19  (0.99-1.43) |  | | 1.71  (1.39-2.11) | |  | 1.11  (0.88-1.40) |  |  |  |
| 45g or greater | 1.22  (0.91-1.64) |  | 2.61  (1.90-3.59) |  | 1.20  (0.80-1.82) |  | | 2.10  (1.35-3.25) | |  | 0.63  (0.32-1.21) |  |  |  |
| **Diabetes** | 1.39  (1.24-1.55) | <0.001 | 1.08  (0.91-1.29) | 0.381 | 1.32  (1.13-1.55) | 0.001 | | 1.03  (0.86-1.24) | | 0.769 | 1.31  (1.07-1.60) | 0.008 | 0.90  (0.66-1.23) | 0.508 |
| **Hypertension** | 1.56  (1.40-1.73) | <0.001 | 1.07  (0.94-1.22) | 0.321 | 1.48  (1.28-1.72) | <0.001 | | 1.05  (0.88-1.26) | | 0.231 | 1.36  (1.13-1.64) | 0.001 | 1.17  (0.93-1.46) | 0.178 |
| **Hyperlipidemia** | 1.31  (1.17-1.47) | <0.001 | 0.98  (0.83-1.08) | 0.839 | 1.25  (1.07-1.47) | 0.005 | | 1.01  (0.80-1.27) | | 0.110 | 1.27  (1.04-1.54) | 0.018 | 1.09  (0.86-1.38) | 0.478 |
| **Ischemic heart disease** | 1.66  (1.36-2.04) | <0.001 | 1.08  (0.86-1.35) | 0.522 | 1.39  (1.05-1.85) | 0.023 | | 0.91  (0.67-1.23) | | 0.037 | 1.28  (0.89-1.83) | 0.187 |  |  |
| **Stroke** | 1.45  (1.08-1.95) | 0.014 | 0.86  (0.62-1.18) | 0.338 | 1.27  (0.84-1.92) | 0.265 | |  | |  | 1.00  (0.57-1.77) | 0.992 |  |  |
| **Kidney disease** | 1.27  (1.09-1.47) | 0.002 | 1.02  (0.87-1.20) | 0.803 | 1.14  (0.92-1.41) | 0.222 | |  | |  | 1.06  (0.81-1.39) | 0.679 |  |  |
| **Liver disease** | 1.21  (1.09-1.34) | <0.001 | 1.14  (1.01-1.28) | 0.034 | 1.14  (0.98-1.31) | 0.088 | |  | |  | 1.22  (1.02-1.46) | 0.029 | 1.17  (0.95-1.42) | 0.133 |

*Male as reference

**^†^**Less than 15g as reference

**Supplementary Table 2.** Association between liver disease severity and the risk (odds ratio [OR]) of overall macular diseases and maculopathy excluding common macular diseases, adjusted for sex, age, and cumulative dose group.

| **Groups** | **Overall macular diseases** | | | | **Maculopathy excluding common macular diseases** | | | |
| --- | --- | --- | --- | --- | --- | --- | --- | --- |
|  | **Univariate** | | **Multivariate** | | **Univariate** | | **Multivariate** | |
|  | **OR (95% CI)** | **P value** | **OR (95% CI)** | **P value** | **OR (95% CI)** | **P value** | **OR (95% CI)** | **P value** |
| No liver disease | 1.0 (ref) | <0.001 | 1.0 (ref) |  | 1.0 (ref) |  | 1.0 (ref) |  |
| Liver disease without liver cirrhosis/failure | 1.30 (1.20-1.40) |  | 1.16 (1.07-1.25) | <0.001 | 1.27 (1.14-1.41) | <0.001 | 1.14 (1.02-1.27) | 0.061 |
| Liver cirrhosis/failure* | 1.67 (1.35-2.07) |  | 1.33 (1.07-1.66) |  | 1.33 (0.98-1.79) |  | 1.08 (0.80-1.47) |  |

CI = confidence interval

*includes ICD-10 codes of K70.2-70.4, K71.1, K71.7, K72, K74.1-74.6, and K76.2
